# 3-(3-Fluoro­phenyl­sulfin­yl)-2,4,5,6-tetra­methyl-1-benzofuran

**DOI:** 10.1107/S1600536811032077

**Published:** 2011-08-11

**Authors:** Pil Ja Seo, Hong Dae Choi, Byeng Wha Son, Uk Lee

**Affiliations:** a Department of Chemistry, Dongeui University, San 24 Kaya-dong Busanjin-gu, Busan 614-714, Republic of Korea; b Department of Chemistry, Pukyong National University, 599-1 Daeyeon 3-dong, Nam-gu, Busan 608-737, Republic of Korea

## Abstract

In the title compound, C_18_H_17_FO_2_S, the 3-fluoro­phenyl ring makes a dihedral angle of 78.60 (5)° with the mean plane of the benzofuran fragment. In the crystal, mol­ecules are linked by weak inter­molecular C—H⋯O hydrogen bonds and weak inter­molecular C—S⋯π [3.490 (2) Å] inter­actions.

## Related literature

For the pharmacological activity of benzofuran compounds, see: Aslam *et al.* (2009 ▶); Galal *et al.* (2009 ▶); Khan *et al.* (2005 ▶). For natural products with benzofuran rings, see: Akgul & Anil (2003 ▶); Soekamto *et al.* (2003 ▶). For structural studies of related 3-(3-fluoro­phenyl­sulfin­yl)-2-methyl-1-benzofuran derivatives, see: Choi *et al.* (2011**a* ▶,b* ▶).
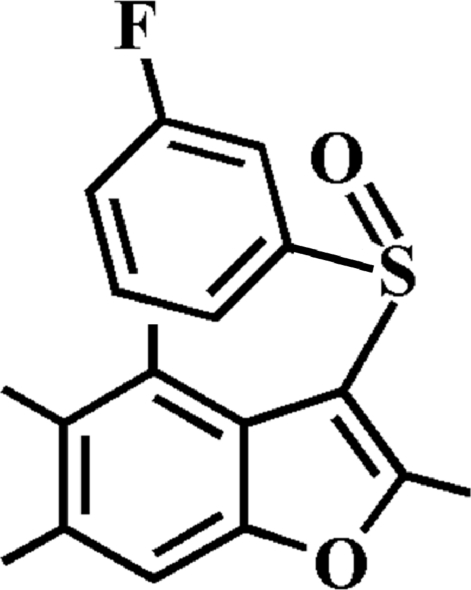


## Experimental

### 

#### Crystal data

C_18_H_17_FO_2_S
*M* *_r_* = 316.38
Triclinic, 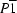

*a* = 8.1631 (13) Å
*b* = 9.2105 (14) Å
*c* = 10.9837 (18) Å
α = 93.089 (9)°
β = 104.812 (9)°
γ = 103.368 (9)°
*V* = 771.1 (2) Å^3^
*Z* = 2
Mo *K*α radiation
μ = 0.23 mm^−1^
*T* = 173 K
0.35 × 0.29 × 0.26 mm

#### Data collection

Bruker SMART APEXII CCD diffractometer
Absorption correction: multi-scan (*SADABS*; Bruker, 2009 ▶) *T*_min_ = 0.926, *T*_max_ = 0.944
13358 measured reflections
3851 independent reflections
2949 reflections with *I* > 2σ(*I*)
*R*_int_ = 0.033

#### Refinement

*R*[*F* ^2^ > 2σ(*F* ^2^)] = 0.046
*wR*(*F* ^2^) = 0.131
*S* = 1.06
3851 reflections
203 parameters
H-atom parameters constrained
Δρ_max_ = 0.49 e Å^−3^
Δρ_min_ = −0.34 e Å^−3^

### 

Data collection: *APEX2* (Bruker, 2009 ▶); cell refinement: *SAINT* (Bruker, 2009 ▶); data reduction: *SAINT*; program(s) used to solve structure: *SHELXS97* (Sheldrick, 2008 ▶); program(s) used to refine structure: *SHELXL97* (Sheldrick, 2008 ▶); molecular graphics: *ORTEP-3* (Farrugia, 1997 ▶) and *DIAMOND* (Brandenburg, 1998 ▶); software used to prepare material for publication: *SHELXL97*.

## Supplementary Material

Crystal structure: contains datablock(s) global, I. DOI: 10.1107/S1600536811032077/vm2116sup1.cif

Structure factors: contains datablock(s) I. DOI: 10.1107/S1600536811032077/vm2116Isup2.hkl

Supplementary material file. DOI: 10.1107/S1600536811032077/vm2116Isup3.cml

Additional supplementary materials:  crystallographic information; 3D view; checkCIF report

## Figures and Tables

**Table 1:** Hydrogen-bond geometry (Å, °)

*D*—H⋯*A*	*D*—H	H⋯*A*	*D*⋯*A*	*D*—H⋯*A*
C14—H14⋯O1^i^	0.95	2.51	3.232 (2)	133
C16—H16⋯O2^ii^	0.95	2.52	3.224 (2)	131

Symmetry codes: (i) 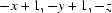; (ii) 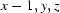.

**Fig. 1.**
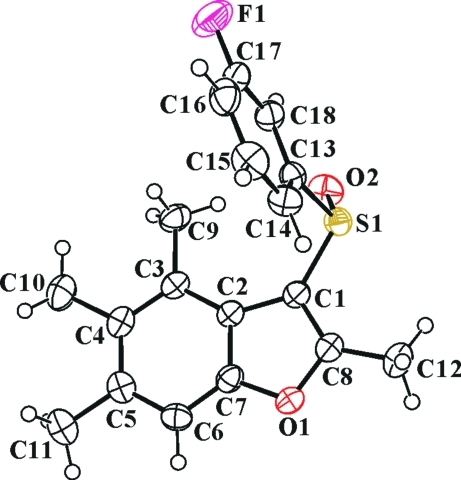
The molecular structure of the title compound with the atom numbering scheme. Displacement ellipsoids are drawn at the 50% probability level. H atoms are presented as a small spheres of arbitrary radius.

**Fig. 2.**
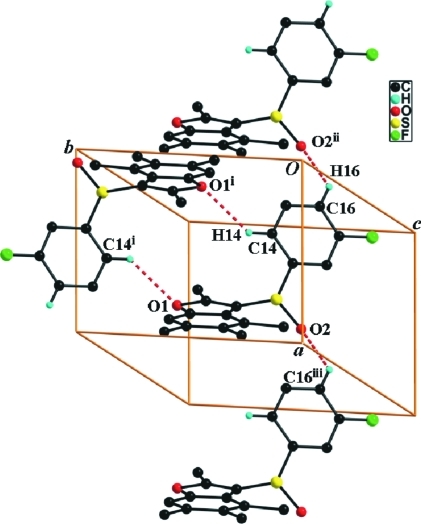
A view of the C—H···O interactions (dotted lines) in the crystal structure of the title compound. [Symmetry codes: (i) - x + 1, - y + 1, - z; (ii) x - 1, y, z; (iii) x + 1, y, z.]

**Fig. 3.**
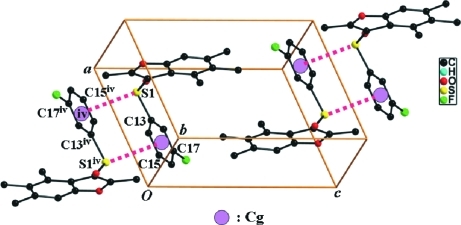
A view of the C—S···π interactions (dotted lines) in the crystal structure of the title compound. [Symmetry codes: (iv) - x + 1, - y, - z .]

## supplementary crystallographic information

### Comment

Many compounds containing a benzofuran ring system have drawn much attention owing to their valuable pharmacological properties such as antibacterial and antifungal, antitumor and antiviral, and antimicrobial activities (Aslam *et al.*, 2009, Galal *et al.*, 2009, Khan *et al.*, 2005). These benzofuran derivatives occur in a wide range of natural products (Akgul & Anil, 2003; Soekamto *et al.*, 2003). As a part of our ongoing study of the substituent effect on the solid state structures of 3-(3-fluorophenylsulfinyl)-2-methyl-1-benzofuran analogues (Choi *et al.*, 2011**a*,b*), we report herein the crystal structure of the title compound.

In the title molecule (Fig. 1), the benzofuran unit is essentially planar, with a mean deviation of 0.018 (1) Å from the least-squares plane defined by the nine constituent atoms. The dihedral angle formed by the 3-fluorophenyl ring and the mean plane of the benzofuran fragment is 78.60 (5)°. The crystal packing (Fig. 2) is stabilized by weak intermolecular C—H···O hydrogen bonds; the first one between a 3-fluorophenyl H atom and the furan O atom (Table 1; C14—H14···O1^i^), and the second one between a 3-fluorophenyl H atom and the O atom of the sulfinyl group (Table 1; C16—H16···O2^ii^). The crystal packing (Fig. 3) is further stabilized by weak intermolecular C—S···π   interactions between the sulfur and 3-fluorophenyl ring, with a C1—S1···Cg^iv^ [3.490 (2) Å] (Cg is the centroid of the C13–C18 3-fluorophenyl ring).

### Experimental

77% 3-chloroperoxybenzoic acid (224 mg, 1.0 mmol) was added in small portions to a stirred solution of 3-(3-fluorophenylsulfanyl)-2,4,5,6-tetramethyl-1-benzofuran (270 mg, 0.8 mmol) in dichloromethane (30 mL) at 273 K. After being stirred at room temperature for 4h, the mixture was washed with saturated sodium bicarbonate solution and the organic layer was separated, dried over magnesium sulfate, filtered and concentrated at reduced pressure. The residue was purified by column chromatography (hexane-ethyl acetate, 2:1 v/v) to afford the title compound as a colorless solid [yield 71%, m.p. 431–432 K; *R*_f_ = 0.57 (hexane–ethyl acetate, 2:1 v/v)]. Single crystals suitable for X-ray diffraction were prepared by slow evaporation of a solution of the title compound in acetone at room temperature.

### Refinement

All H atoms were positioned geometrically and refined using a riding model, with C—H = 0.95 Å for aryl and 0.98 Å for methyl H atoms. Uiso(H) = 1.2*U*_eq_(C)  for aryl and 1.50*U*_eq_(C) for methyl H atoms.

### Crystal data

**Table:**

C_18_H_17_FO_2_S	*Z* = 2
*M**_r_* = 316.38	*F*(000) = 332
Triclinic, *P*1	*D*_x_ = 1.363 Mg m^−^^3^
Hall symbol: -P 1	Mo *K*α radiation, λ = 0.71073 Å
*a* = 8.1631 (13) Å	Cell parameters from 4936 reflections
*b* = 9.2105 (14) Å	θ = 2.8–27.9°
*c* = 10.9837 (18) Å	µ = 0.23 mm^−^^1^
α = 93.089 (9)°	*T* = 173 K
β = 104.812 (9)°	Block, colourless
γ = 103.368 (9)°	0.35 × 0.29 × 0.26 mm
*V* = 771.1 (2) Å^3^	

### Data collection

**Table:**

Bruker SMART APEXII CCD diffractometer	3851 independent reflections
Radiation source: rotating anode	2949 reflections with *I* > 2σ(*I*)
graphite multilayer	*R*_int_ = 0.033
Detector resolution: 10.0 pixels mm^-1^	θ_max_ = 28.5°, θ_min_ = 1.9°
φ and ω scans	*h* = −10→10
Absorption correction: multi-scan (*SADABS*; Bruker, 2009)	*k* = −12→12
*T*_min_ = 0.926, *T*_max_ = 0.944	*l* = −14→14
13358 measured reflections	

### Refinement

**Table:**

Refinement on *F*^2^	Primary atom site location: structure-invariant direct methods
Least-squares matrix: full	Secondary atom site location: difference Fourier map
*R*[*F*^2^ > 2σ(*F*^2^)] = 0.046	Hydrogen site location: difference Fourier map
*wR*(*F*^2^) = 0.131	H-atom parameters constrained
*S* = 1.06	*w* = 1/[σ^2^(*F*_o_^2^) + (0.0623*P*)^2^ + 0.2521*P*] where *P* = (*F*_o_^2^ + 2*F*_c_^2^)/3
3851 reflections	(Δ/σ)_max_ < 0.001
203 parameters	Δρ_max_ = 0.49 e Å^−^^3^
0 restraints	Δρ_min_ = −0.34 e Å^−^^3^

### Special details

**Table:**

Geometry. All esds (except the esd in the dihedral angle between two l.s. planes) are estimated using the full covariance matrix. The cell esds are taken into account individually in the estimation of esds in distances, angles and torsion angles; correlations between esds in cell parameters are only used when they are defined by crystal symmetry. An approximate (isotropic) treatment of cell esds is used for estimating esds involving l.s. planes.
Refinement. Refinement of F^2^ against ALL reflections. The weighted R-factor wR and goodness of fit S are based on F^2^, conventional R-factors R are based on F, with F set to zero for negative F^2^. The threshold expression of F^2^ > 2sigma(F^2^) is used only for calculating R-factors(gt) etc. and is not relevant to the choice of reflections for refinement. R-factors based on F^2^ are statistically about twice as large as those based on F, and R- factors based on ALL data will be even larger.

### Fractional atomic coordinates and isotropic or equivalent isotropic displacement parameters (Å^2^)

**Table:**

	*x*	*y*	*z*	*U*_iso_*/*U*_eq_	
S1	0.72575 (6)	0.17448 (5)	0.11804 (5)	0.03550 (15)	
O1	0.79125 (18)	0.60446 (14)	0.10097 (13)	0.0397 (3)	
O2	0.84594 (17)	0.11260 (15)	0.21415 (15)	0.0468 (4)	
F1	0.2895 (2)	−0.15422 (17)	0.30881 (14)	0.0714 (4)	
C1	0.7609 (2)	0.36974 (19)	0.15447 (18)	0.0326 (4)	
C2	0.7692 (2)	0.46866 (18)	0.26657 (17)	0.0308 (4)	
C3	0.7630 (2)	0.45329 (19)	0.39109 (17)	0.0328 (4)	
C4	0.7669 (2)	0.5822 (2)	0.46760 (18)	0.0373 (4)	
C5	0.7842 (2)	0.7238 (2)	0.42159 (19)	0.0383 (4)	
C6	0.7966 (2)	0.7392 (2)	0.29960 (19)	0.0376 (4)	
H6	0.8108	0.8340	0.2681	0.045*	
C7	0.7872 (2)	0.6100 (2)	0.22591 (17)	0.0339 (4)	
C8	0.7739 (3)	0.4571 (2)	0.06027 (19)	0.0378 (4)	
C9	0.7545 (3)	0.3039 (2)	0.44198 (19)	0.0435 (5)	
H9A	0.6354	0.2603	0.4472	0.065*	
H9B	0.8369	0.3183	0.5267	0.065*	
H9C	0.7858	0.2359	0.3851	0.065*	
C10	0.7545 (3)	0.5700 (3)	0.6016 (2)	0.0526 (5)	
H10A	0.8724	0.5994	0.6605	0.079*	
H10B	0.6979	0.4661	0.6090	0.079*	
H10C	0.6852	0.6368	0.6222	0.079*	
C11	0.7909 (3)	0.8623 (2)	0.5049 (2)	0.0516 (5)	
H11A	0.8131	0.9508	0.4601	0.077*	
H11B	0.8852	0.8748	0.5835	0.077*	
H11C	0.6788	0.8511	0.5251	0.077*	
C12	0.7644 (3)	0.4258 (3)	−0.0756 (2)	0.0547 (6)	
H12A	0.7929	0.3297	−0.0900	0.082*	
H12B	0.8484	0.5064	−0.0988	0.082*	
H12C	0.6458	0.4205	−0.1278	0.082*	
C13	0.5135 (2)	0.11544 (18)	0.14512 (17)	0.0316 (4)	
C14	0.3797 (2)	0.1754 (2)	0.0829 (2)	0.0395 (4)	
H14	0.4004	0.2516	0.0297	0.047*	
C15	0.2153 (3)	0.1225 (2)	0.0995 (2)	0.0475 (5)	
H15	0.1225	0.1637	0.0578	0.057*	
C16	0.1836 (3)	0.0112 (2)	0.1754 (2)	0.0467 (5)	
H16	0.0703	−0.0248	0.1869	0.056*	
C17	0.3193 (3)	−0.0457 (2)	0.23361 (19)	0.0423 (5)	
C18	0.4854 (2)	0.0028 (2)	0.22066 (18)	0.0373 (4)	
H18	0.5773	−0.0396	0.2621	0.045*	

### Atomic displacement parameters (Å^2^)

**Table:**

	*U*^11^	*U*^22^	*U*^33^	*U*^12^	*U*^13^	*U*^23^
S1	0.0347 (3)	0.0348 (2)	0.0412 (3)	0.01320 (17)	0.0138 (2)	0.00494 (18)
O1	0.0531 (8)	0.0371 (6)	0.0327 (7)	0.0133 (6)	0.0154 (6)	0.0119 (5)
O2	0.0366 (8)	0.0456 (7)	0.0624 (10)	0.0214 (6)	0.0101 (7)	0.0107 (7)
F1	0.0838 (10)	0.0742 (9)	0.0614 (10)	0.0083 (8)	0.0357 (8)	0.0279 (7)
C1	0.0318 (9)	0.0349 (8)	0.0336 (10)	0.0104 (7)	0.0110 (7)	0.0079 (7)
C2	0.0281 (9)	0.0345 (8)	0.0320 (9)	0.0106 (7)	0.0088 (7)	0.0083 (7)
C3	0.0281 (9)	0.0390 (9)	0.0311 (10)	0.0087 (7)	0.0064 (7)	0.0092 (7)
C4	0.0315 (9)	0.0481 (10)	0.0319 (10)	0.0097 (8)	0.0085 (8)	0.0062 (8)
C5	0.0332 (10)	0.0421 (9)	0.0378 (11)	0.0117 (7)	0.0059 (8)	0.0003 (8)
C6	0.0375 (10)	0.0340 (8)	0.0418 (11)	0.0118 (7)	0.0086 (8)	0.0084 (8)
C7	0.0348 (9)	0.0391 (9)	0.0303 (10)	0.0126 (7)	0.0094 (7)	0.0101 (7)
C8	0.0414 (10)	0.0394 (9)	0.0353 (10)	0.0100 (8)	0.0148 (8)	0.0074 (8)
C9	0.0498 (12)	0.0447 (10)	0.0348 (11)	0.0090 (9)	0.0100 (9)	0.0140 (8)
C10	0.0584 (14)	0.0648 (13)	0.0352 (12)	0.0138 (11)	0.0156 (10)	0.0055 (10)
C11	0.0556 (13)	0.0496 (11)	0.0490 (13)	0.0177 (10)	0.0117 (10)	−0.0052 (10)
C12	0.0732 (16)	0.0562 (12)	0.0380 (12)	0.0125 (11)	0.0243 (11)	0.0084 (10)
C13	0.0321 (9)	0.0322 (8)	0.0314 (9)	0.0108 (7)	0.0080 (7)	0.0023 (7)
C14	0.0364 (10)	0.0382 (9)	0.0441 (11)	0.0140 (7)	0.0065 (8)	0.0086 (8)
C15	0.0349 (11)	0.0521 (11)	0.0553 (14)	0.0178 (9)	0.0066 (9)	0.0040 (10)
C16	0.0370 (11)	0.0538 (11)	0.0494 (13)	0.0074 (9)	0.0176 (9)	−0.0037 (10)
C17	0.0529 (12)	0.0418 (10)	0.0329 (10)	0.0066 (8)	0.0181 (9)	0.0045 (8)
C18	0.0417 (11)	0.0391 (9)	0.0332 (10)	0.0147 (8)	0.0092 (8)	0.0080 (8)

### Geometric parameters (Å, °)

**Table:**

S1—O2	1.4818 (14)	C9—H9C	0.9800
S1—C1	1.7613 (17)	C10—H10A	0.9800
S1—C13	1.7975 (18)	C10—H10B	0.9800
O1—C8	1.369 (2)	C10—H10C	0.9800
O1—C7	1.380 (2)	C11—H11A	0.9800
F1—C17	1.349 (2)	C11—H11B	0.9800
C1—C8	1.355 (3)	C11—H11C	0.9800
C1—C2	1.469 (2)	C12—H12A	0.9800
C2—C7	1.387 (2)	C12—H12B	0.9800
C2—C3	1.395 (2)	C12—H12C	0.9800
C3—C4	1.406 (3)	C13—C18	1.378 (3)
C3—C9	1.507 (3)	C13—C14	1.379 (2)
C4—C5	1.414 (3)	C14—C15	1.380 (3)
C4—C10	1.509 (3)	C14—H14	0.9500
C5—C6	1.382 (3)	C15—C16	1.376 (3)
C5—C11	1.511 (3)	C15—H15	0.9500
C6—C7	1.378 (2)	C16—C17	1.362 (3)
C6—H6	0.9500	C16—H16	0.9500
C8—C12	1.484 (3)	C17—C18	1.373 (3)
C9—H9A	0.9800	C18—H18	0.9500
C9—H9B	0.9800		
			
O2—S1—C1	111.56 (8)	C4—C10—H10B	109.5
O2—S1—C13	106.31 (9)	H10A—C10—H10B	109.5
C1—S1—C13	98.37 (8)	C4—C10—H10C	109.5
C8—O1—C7	106.59 (14)	H10A—C10—H10C	109.5
C8—C1—C2	107.14 (15)	H10B—C10—H10C	109.5
C8—C1—S1	117.83 (14)	C5—C11—H11A	109.5
C2—C1—S1	134.88 (14)	C5—C11—H11B	109.5
C7—C2—C3	119.02 (16)	H11A—C11—H11B	109.5
C7—C2—C1	103.97 (15)	C5—C11—H11C	109.5
C3—C2—C1	137.01 (16)	H11A—C11—H11C	109.5
C2—C3—C4	117.74 (16)	H11B—C11—H11C	109.5
C2—C3—C9	120.95 (16)	C8—C12—H12A	109.5
C4—C3—C9	121.30 (17)	C8—C12—H12B	109.5
C3—C4—C5	121.10 (17)	H12A—C12—H12B	109.5
C3—C4—C10	119.64 (18)	C8—C12—H12C	109.5
C5—C4—C10	119.26 (18)	H12A—C12—H12C	109.5
C6—C5—C4	120.82 (17)	H12B—C12—H12C	109.5
C6—C5—C11	118.33 (18)	C18—C13—C14	121.52 (17)
C4—C5—C11	120.85 (19)	C18—C13—S1	118.72 (14)
C7—C6—C5	116.64 (17)	C14—C13—S1	119.58 (14)
C7—C6—H6	121.7	C13—C14—C15	118.69 (19)
C5—C6—H6	121.7	C13—C14—H14	120.7
C6—C7—O1	124.15 (16)	C15—C14—H14	120.7
C6—C7—C2	124.60 (17)	C16—C15—C14	121.15 (19)
O1—C7—C2	111.23 (15)	C16—C15—H15	119.4
C1—C8—O1	111.06 (16)	C14—C15—H15	119.4
C1—C8—C12	133.92 (18)	C17—C16—C15	118.01 (19)
O1—C8—C12	114.94 (17)	C17—C16—H16	121.0
C3—C9—H9A	109.5	C15—C16—H16	121.0
C3—C9—H9B	109.5	F1—C17—C16	118.40 (19)
H9A—C9—H9B	109.5	F1—C17—C18	118.29 (19)
C3—C9—H9C	109.5	C16—C17—C18	123.3 (2)
H9A—C9—H9C	109.5	C17—C18—C13	117.31 (18)
H9B—C9—H9C	109.5	C17—C18—H18	121.3
C4—C10—H10A	109.5	C13—C18—H18	121.3
			
O2—S1—C1—C8	132.60 (15)	C8—O1—C7—C2	0.6 (2)
C13—S1—C1—C8	−116.10 (16)	C3—C2—C7—C6	−1.2 (3)
O2—S1—C1—C2	−52.5 (2)	C1—C2—C7—C6	178.53 (17)
C13—S1—C1—C2	58.80 (19)	C3—C2—C7—O1	179.95 (15)
C8—C1—C2—C7	−0.08 (19)	C1—C2—C7—O1	−0.31 (19)
S1—C1—C2—C7	−175.35 (15)	C2—C1—C8—O1	0.4 (2)
C8—C1—C2—C3	179.6 (2)	S1—C1—C8—O1	176.66 (12)
S1—C1—C2—C3	4.3 (3)	C2—C1—C8—C12	−176.0 (2)
C7—C2—C3—C4	3.0 (2)	S1—C1—C8—C12	0.3 (3)
C1—C2—C3—C4	−176.65 (18)	C7—O1—C8—C1	−0.6 (2)
C7—C2—C3—C9	−176.26 (17)	C7—O1—C8—C12	176.51 (17)
C1—C2—C3—C9	4.1 (3)	O2—S1—C13—C18	−14.81 (17)
C2—C3—C4—C5	−2.6 (3)	C1—S1—C13—C18	−130.29 (15)
C9—C3—C4—C5	176.59 (18)	O2—S1—C13—C14	169.93 (14)
C2—C3—C4—C10	178.03 (17)	C1—S1—C13—C14	54.46 (16)
C9—C3—C4—C10	−2.7 (3)	C18—C13—C14—C15	1.3 (3)
C3—C4—C5—C6	0.4 (3)	S1—C13—C14—C15	176.42 (15)
C10—C4—C5—C6	179.75 (18)	C13—C14—C15—C16	−0.6 (3)
C3—C4—C5—C11	−179.15 (17)	C14—C15—C16—C17	−0.2 (3)
C10—C4—C5—C11	0.2 (3)	C15—C16—C17—F1	179.61 (18)
C4—C5—C6—C7	1.4 (3)	C15—C16—C17—C18	0.3 (3)
C11—C5—C6—C7	−179.02 (17)	F1—C17—C18—C13	−178.93 (17)
C5—C6—C7—O1	177.67 (17)	C16—C17—C18—C13	0.4 (3)
C5—C6—C7—C2	−1.0 (3)	C14—C13—C18—C17	−1.2 (3)
C8—O1—C7—C6	−178.27 (17)	S1—C13—C18—C17	−176.35 (13)

### Hydrogen-bond geometry (Å, °)

**Table:**

*D*—H···*A*	*D*—H	H···*A*	*D*···*A*	*D*—H···*A*
C14—H14···O1^i^	0.95	2.51	3.232 (2)	133.
C16—H16···O2^ii^	0.95	2.52	3.224 (2)	131.

Symmetry codes: (i) −*x*+1, −*y*+1, −*z*; (ii) *x*−1, *y*, *z*.
